# Thioredoxin A Active-Site Mutants Form Mixed Disulfide Dimers That Resemble Enzyme–Substrate Reaction Intermediates

**DOI:** 10.1016/j.jmb.2008.03.077

**Published:** 2008-06-06

**Authors:** Thijs R.H.M. Kouwen, Juni Andréll, Rianne Schrijver, Jean-Yves F. Dubois, Megan J. Maher, So Iwata, Elisabeth P. Carpenter, Jan Maarten van Dijl

**Affiliations:** 1 Department of Medical Microbiology, University Medical Center Groningen and University of Groningen, Hanzeplein 1, PO Box 30001, 9700 RB Groningen, The Netherlands; 2 Department of Biological Sciences, Imperial College of Science, Technology and Medicine, Exhibition Road, South Kensington, London SW7 2AZ, UK

**Keywords:** disulfide, thioredoxin, *Bacillus*, structure, dimer, TrxA, thioredoxin A, BsTrxA, *Bacillus subtilis* TrxA, ArsC, arsenate reductase, BASI, barley α-amylase/subtilisin inhibitor, PDB, Protein Data Bank, AMS, 4-acetamido-4′-maleimidyl-stilbene-2,2′-disulfonate, NF-κB, nuclear factor κB, ESRF, European Synchrotron Radiation Facility

## Abstract

Thioredoxin functions in nearly all organisms as the major thiol–disulfide oxidoreductase within the cytosol. Its prime purpose is to maintain cysteine-containing proteins in the reduced state by converting intramolecular disulfide bonds into dithiols in a disulfide exchange reaction. Thioredoxin has been reported to contribute to a wide variety of physiological functions by interacting with specific sets of substrates in different cell types. To investigate the function of the essential thioredoxin A (TrxA) in the low-GC Gram-positive bacterium *Bacillus subtilis*, we purified wild-type TrxA and three mutant TrxA proteins that lack either one or both of the two cysteine residues in the CxxC active site. The pure proteins were used for substrate-binding studies known as “mixed disulfide fishing” in which covalent disulfide-bonded reaction intermediates can be visualized. An unprecedented finding is that both active-site cysteine residues can form mixed disulfides with substrate proteins when the other active-site cysteine is absent, but only the N-terminal active-site cysteine forms stable interactions. A second novelty is that both single-cysteine mutant TrxA proteins form stable homodimers due to thiol oxidation of the remaining active-site cysteine residue. To investigate whether these dimers resemble mixed enzyme–substrate disulfides, the structure of the most abundant dimer, C32S, was characterized by X-ray crystallography. This yielded a high-resolution (1.5Å) X-ray crystallographic structure of a thioredoxin homodimer from a low-GC Gram-positive bacterium. The C32S TrxA dimer can be regarded as a mixed disulfide reaction intermediate of thioredoxin, which reveals the diversity of thioredoxin/substrate-binding modes.

## Introduction

Thioredoxin is a ubiquitous protein that is present in nearly all known organisms from archaea to humans.^1^ It is a ∼ 12-kDa soluble protein with a characteristic thioredoxin fold, which is active in the cytosol. Its prime purpose is to maintain cysteine-containing proteins in the reduced state by converting intramolecular disulfide bonds into dithiols. Thioredoxin thus has an important thiol–disulfide oxidoreductase function in the cell, and this is the main reason that this enzyme has been studied extensively since it was first described in 1964.^2^ Additionally, it has been proposed that thioredoxins have chaperone activity in protein folding.^3^ The majority of these studies have been conducted on *Escherichia coli* thioredoxin A (TrxA) and human thioredoxin 1. The latter enzyme, hereinafter referred to as Trx1, has a role in the regulation of cell proliferation and differentiation and in programmed cell death. Hence, it is a key target for the development of drugs to treat cancer, especially since increased thioredoxin levels are seen in many human primary cancers.^4^ In plant chloroplasts, thioredoxin regulates the light-activated Calvin cycle;^5^ in *E. coli*, thioredoxin plays a critical role in cellular responses to oxidative stress.^6^ In general, the specific physiological roles of thioredoxins in different organisms are directly related to the specific functions of their substrates in these organisms.

In its active site, thioredoxin has two cysteine residues in a conserved amino acid motif Trp-Cys-Gly-Pro-Cys, which is a defining characteristic of thioredoxins.^7^ The generally believed mode of action of thioredoxins is as follows.^8–10^ An oxidized disulfide-containing substrate protein will bind to a conserved hydrophobic surface of the thioredoxin. In the active form, the catalytic cysteines of thioredoxin are reduced. Importantly, the lower p*K*_a_ value of the N-terminal cysteine of thioredoxin promotes its existence as a thiolate, the reactive deprotonated form of thiol, and this thiolate can act as a nucleophile to combine with a protein substrate, resulting in a covalently linked mixed disulfide (thioredoxin–*S*–*S*–protein). The close proximity (i.e., high local concentration) of the thiol group of the second C-terminal cysteine in the thioredoxin active site facilitates the next step of the reaction where the fully reduced protein and the disulfide-containing thioredoxin are generated. Oxidized thioredoxin is then reduced back to its active form by thioredoxin reductase.^11^

The three-dimensional structures of many thioredoxin variants have been determined to visualize the protein states during specific stages of the thioredoxin catalytic cycle. The structure of the wild-type *Bacillus subtilis* TrxA (BsTrxA) was originally solved by NMR as a monomer in the oxidized and reduced states.^12^ The structure of the BsTrxA C32S mutant was solved by NMR as a heterodimer complex with the substrate protein, arsenate reductase (ArsC).^12^ In addition, there are structures for uncomplexed human Trx1, which has been structurally characterized in both its reduced state and its oxidized state by X-ray crystallography,^13^ and with bound peptides from substrate proteins by NMR.^14,15^ There is also a structure for the plant thioredoxin/substrate complex between barley thioredoxin and α-amylase/subtilisin inhibitor [barley α-amylase/subtilisin inhibitor (BASI)].^16^ The *E. coli* thioredoxins have similarly been structurally characterized in detail.^17,18^ Several other uncomplexed thioredoxin structures, from humans, *E. coli*, and other sources, are available in the Protein Data Bank (PDB; summarized in Maeda *et al.*^16^). The interaction between thioredoxin and thioredoxin reductase, the enzyme that reduces thioredoxin to restore it to its active state, has been visualized in structures of the complexes between the *E. coli* thioredoxin reductase and thioredoxin,^19^ and also between ferrodoxin–thioredoxin reductase and spinach chloroplast thioredoxins (Trx-f and Trx-m).^20^ In addition, there is a 2.2-Å crystal structure of thioredoxin in complex with DNA polymerase from bacteriophage T7.^21^ In this study, however, thioredoxin forms a stable complex involved in the processivity of DNA replication for which its redox function is not required. The wealth of available structures allows comparison of complex structures from which more general information can be derived.

In contrast to its counterparts from humans, plant chloroplasts, and Gram-negative bacteria such as *E. coli*, relatively little is known about the TrxA of Gram-positive bacteria such as *B. subtilis.* It has been shown that the *trxA* gene is essential for the growth and viability of *B. subtilis*, and its expression at elevated levels is triggered by multiple stresses.^22,23^ Furthermore, TrxA limitation represents a form of stress that impacts on a wide range of physiologically and developmentally important processes in *B. subtilis*, such as the development of competence for DNA binding and uptake.^24^ The essentiality of *trxA* in *B. subtilis* underpins a remarkable biological difference from the situation in *E. coli* where the two genes for thioredoxins (*trxA* and *trxC*) are not essential.^25^ Most likely, this difference relates to the absence of glutathione from *B. subtilis*^26^ and the presence of this reducing agent in *E. coli*.^11,27^ This difference focuses interest on the substrates of TrxA in *B. subtilis*, some of which will be essential for growth and viability, as argued above. A highly elegant technique, named mixed disulfide fishing, has been employed to identify thioredoxin/substrate in various biological systems.^28,29^ In essence, the second C-terminal cysteine in the thioredoxin active site is replaced with serine. The resulting mutant thioredoxin is still able to bind its substrates via the N-terminal (attacking) cysteine, but the subsequent release of a reduced substrate is precluded by the absence of the C-terminal (resolving) cysteine. Consequently, normally very short-lived covalent enzyme–substrate reaction intermediates accumulate, which can then be purified and analyzed to identify the bound substrates.

In the present study, we have established the tools for mixed disulfide fishing in *B. subtilis*, including highly purified active-site mutant BsTrxA proteins (i.e., C29S and C32S single and double mutants) and a wild-type TrxA control. Our special attention was focused on the characterization of the single-cysteine mutant proteins because both mutant proteins displayed a so far uncharacterized auto-oxidation that resulted in homodimer formation under nonreducing conditions. Consistent with our findings, early studies by Chandrasekhar *et al.*, Dyson *et al.*, Jeng *et al.*, and Russel *et al.* on thioredoxins of *E. coli* mention that DTT-reducible dimers can be formed by active-site mutant proteins lacking the resolving cysteine, but the respective observations were not documented in detail.^30–33^ Furthermore, the homodimer formation of thioredoxin mutant proteins lacking the attacking cysteine was unprecedented. To define the molecular basis of these observations, we set out to characterize the three-dimensional structure of the C32S BsTrxA homodimer by X-ray crystallography. Here we present the first structure of a BsTrxA C32S homodimer at 1.5 Å resolution. Comparison of this structure with the NMR structure of the BsTrxA complexed with a substrate, ArsC, shows two distinct modes of substrate binding in which the peptide chains are bound in different orientations. There is a conserved hydrophobic pocket on one side of the catalytic N-terminal cysteine, which binds a large hydrophobic residue in many thioredoxin structures.

## Results

### Mixed disulfide fishing

**Fig. 1:**
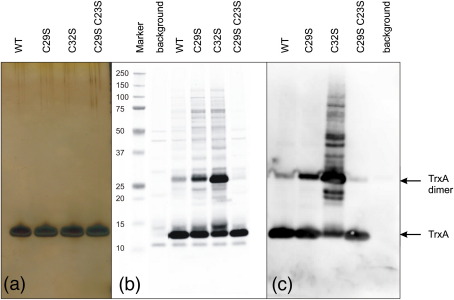
Mixed disulfide fishing. Mixed disulfide fishing was performed with the highly pure His6-tagged BsTrxA with the wild-type active site or with active-site-specific mutations, and cytoplasmic proteins from the TrxA-depleted *B. subtilis* WB800 I*trxA* strain. (a) To show that the BsTrxA proteins used for mixed disulfide fishing were highly pure, 0.1 μg of each BsTrxA protein variant was loaded on SDS-PAGE. Upon electrophoresis, the gel was silver-stained. WT, BsTrxA with wild-type active site; C29S, C29S single-mutant BsTrxA; C32S, C32S single-mutant BsTrxA; C29S–C33S, C29S–C32S double-mutant BsTrxA. (b) To visualize possible stable interactions between BsTrxA and its substrates, 2 μg of each BsTrxA protein variant was mixed with 50 μl of cytoplasmic protein extract. After 5 min of incubation, proteins were separated on a nonreducing gel, and BsTrxA–substrate complexes were visualized by Western blotting, with antibodies raised against *B. subtilis* TrxA. Background: The cytoplasmic extract mock-treated with 2 μl of water instead of BsTrxA protein reveals a few nonspecific cross-reactions of the BsTrxA antibody. (c) Immunodetection of purified BsTrxA–substrate complexes. For purification of the BsTrxA–substrate bound complexes, the C-terminal His6-tag of the pure BsTrxA proteins was used. Pure BsTrxA with the wild type or a mutant active site was mixed with cytoplasmic protein extracts as indicated for (b). Subsequently, magnetic beads precharged with nickel were added, and the His6-tag of the BsTrxA proteins was allowed to bind to the nickel of the magnetic beads for 10 min. A magnet was then used to collect the beads with bound BsTrxA. After the beads had been washed nine times, the BsTrxA proteins were eluted from the beads with a buffer containing imidazole. The eluted proteins were separated by nonreducing SDS-PAGE, and the BsTrxA–substrate bound complexes were visualized by Western blotting with antibodies against BsTrxA.

As a first approach to developing the tools for mixed disulfide fishing of potential substrates of BsTrxA, we introduced single-cysteine mutations (i.e., C29S or C32S) or a double-cysteine mutation (C29S–C32S) into this protein. In addition, wild-type BsTrxA and the three cysteine-mutant derivatives were provided with a C-terminal His6-tag, allowing their successful purification to ∼1mg/ml by Ni-affinity chromatography. A silver-stained SDS-poly acryl amide gel of these purified proteins shows that the respective samples did not contain any detectable impurities (Fig. 1a), and this was confirmed by mass spectrometry (data not shown). Next we tested whether these four purified proteins would bind to cytoplasmic proteins of a *B. subtilis* strain that was depleted for TrxA. The rationale behind this experiment was that potential oxidized TrxA substrate proteins would be present at elevated levels in a strain depleted for TrxA. To probe their interaction with potential substrates, the four purified BsTrxA variants (wild type, C29S, C32S, and the double mutant) were mixed with the cytoplasmic proteins, incubated for 5min at room temperature, and subsequently separated by SDS-PAGE under nonreducing conditions. Next, the presence of stable TrxA–substrate reaction intermediates, which would include mixed disulfides, was analyzed by Western blotting using antibodies against BsTrxA. The results of these interaction studies are shown in Fig. 1b. As expected, it was found that the C32S BsTrxA, which contains the attacking Cys29 residue, was able to form stable interactions with potential substrate proteins, whereas the C29S–C32S double mutant did not form these interactions. This observation is fully consistent with the known reaction mechanism of thioredoxins and the anticipated accumulation of enzyme–substrate complexes when the resolving Cys32 is absent. In the experiment with the double-mutant C29S–C32S, only a few bands of cytoplasmic proteins that cross-reacted with the TrxA antibody were detectable (Fig. 1b; compare lanes labeled “background” and “C29S–C32S”). Similar to the results obtained with C32S BsTrxA, enzyme–substrate complexes were identified when the wild-type TrxA was incubated with potential substrates—consistent with the fact that the catalytic site of this purified protein is intact. Quite unexpectedly, however, we observed that the C29S BsTrxA variant (lacking the attacking Cys29 residue) was also able to form reaction intermediates that could be separated by SDS-PAGE, albeit with a slightly lower efficiency than the C32S BsTrxA (Fig. 1b). Importantly, the amount of reaction intermediates accumulating with the C29S protein was much higher than that observed for the wild-type BsTrxA protein (Fig. 1b). This suggested that Cys32 can act as a nucleophile in BsTrxA(C29S)–substrate interactions.

To further test the stability of the observed reaction intermediates, we made use of the nickel-binding property of the C-terminal His6-tag of the purified BsTrxA proteins in an assay where binding to magnetic beads precharged with nickel was analyzed. Upon incubation of purified wild-type or mutant BsTrxA proteins with potential substrates and these magnetic beads, the resulting substrate–BsTrxA–beads complexes were extracted from the reaction mixtures using a magnet. The results of these “mixed disulfide fishing” experiments are depicted in Fig. 1c. This figure clearly shows that only the mixed disulfides formed by the C32S BsTrxA mutant protein were sufficiently stable for fishing. In contrast, all reaction intermediates with the C29S mutant or the wild-type BsTrxA protein were lost during the washing of the beads (Fig. 1c), implying that the respective interactions were significantly less stable than those observed for the C32S mutant and its potential substrates.

Notably, the interaction analyses indicated that all purified TrxA proteins were capable of dimer formation when analyzed under nonreducing conditions, as evidenced by the presence of bands with twice the mass of monomeric TrxA (Fig. 1b and c). Such potential dimers were present when purified TrxA proteins were mixed with potential substrates, and they remained stable upon mixed disulfide fishing with magnetic beads. Furthermore, the largest amounts of potential dimers were detected for the single-cysteine mutant proteins, the C32S mutant in particular. By contrast, dimers of the double-cysteine mutant protein were barely detectable. Both the finding that the C29S mutant protein was apparently able to bind potential substrates and the observed significant levels of dimerization of both single-cysteine mutant BsTrxA proteins prompted us to focus further attention on the properties of the single-cysteine mutant BsTrxA proteins.

### Redox state of BsTrxA

**Fig. 2:**
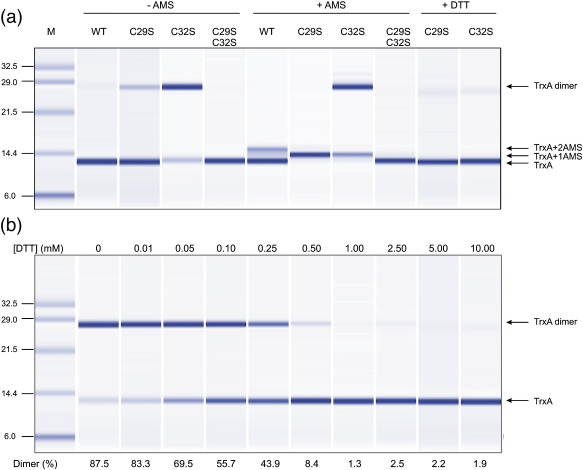
Redox states of BsTrxA monomers and dimers. (a) Purified His6-tagged BsTrxA proteins were separated by capillary electrophoresis using a 2100 Bioanalyzer (Agilent Technologies). WT, BsTrxA with wild-type active site; C29S, C29S single-mutant BsTrxA; C32S, C32S single-mutant BsTrxA; C29S–C32S, C29S–C32S double-mutant BsTrxA. To monitor the presence of free thiols in the purified BsTrxA proteins, samples were incubated in the presence or in the absence of 0.3 mM AMS (lanes marked + AMS or − AMS). To test whether the C29S and C32S dimers are formed by disulfide bonding, these proteins were incubated with 10 mM DTT (lanes marked + DTT). DTT was absent from all other samples. The image of the Bioanalyzer chromatogram was generated using the 2100 Expert Software package (Agilent Technologies). (b) C32S BsTrxA protein (∼ 2.5 μg) was reduced with increasing concentrations of DTT (shown on top of the panel) and separated by capillary electrophoresis as described for (a). The dimer-to-monomer ratios (Dimer [%]) are shown at the bottom of the panel.

To study the properties of the C29S and C32S single-mutant BsTrxA protein variants, the purified His-tagged proteins were analyzed both by standard SDS-PAGE and by capillary electrophoresis with a 2100 Bioanalyzer (Agilent Technologies) under reducing and nonreducing conditions. The results obtained in both approaches were highly comparable and, therefore, only the Bioanalyzer results are shown in Fig. 2. Under nonreducing conditions, the pure wild-type BsTrxA protein and the C29S–C32S double-mutant protein showed an electrophoretic mobility that was in accordance with the molecular weight of BsTrxA (∼12 kDa). As already observed in the mixed disulfide fishing experiments, the pure C29S and C32S single-mutant proteins were separated as two species under nonreducing conditions, one of which migrated at ∼12 kDa whereas the other migrated at ∼25 kDa. As confirmed by Western blotting using antibodies against BsTrxA, the ∼25-kDa band consisted of BsTrxA (not shown). Notably, the relative abundance of the ∼25-kDa species was higher for the C32S BsTrxA mutant protein (∼81%) than for the C29S BsTrxA mutant protein (∼17%). Furthermore, the band of ∼25 kDa was not observed in the presence of the reducing agent DTT (Fig. 2a). Together, these observations suggested that the 25-kDa band represents a BsTrxA dimer in which the two identical subunits are held together by a bond that can be broken with DTT. In order to estimate the quantity of DTT required for the breakage of this bond, the C32S BsTrxA protein was exposed to increasing concentrations of DTT. As shown in Fig. 2b, about 0.5 mM DTT was required for the reduction of most C32S BsTrxA homodimers to monomers, and about 0.15–0.2 mM DTT was required to reduce 50% of these dimers. These observations imply that the dimerization of the single-cysteine mutant proteins was due to disulfide bond formation between the active-site cysteine residues of two monomers, firstly because Cys29 and Cys32 are the only two-cysteine residues in BsTrxA and, secondly, because no stable dimers were detectable for the C29S–C32S double mutant (Fig. 2a). To verify this idea, cross-linking experiments were performed with the thiol-specific reagent 4-acetamido-4′-maleimidyl-stilbene-2,2′-disulfonate (AMS). The binding of AMS is known to result in a decreased electrophoretic mobility of ∼0.5 kDa per bound AMS molecule, allowing the separation of reduced and oxidized proteins.^34^ As shown in Fig. 2a, AMS-treated wild-type BsTrxA molecules migrated in two separate bands of ∼12 and ∼13 kDa, representing oxidized TrxA (lower band) and reduced BsTrxA with two bound AMS molecules (upper band), respectively. By peak integration, it was estimated that only about 33% of the wild-type BsTrxA was in a reduced state under the conditions tested. As expected, the cysteine-free C29S–C32S double-mutant BsTrxA protein did not bind AMS (Fig. 2a). The monomeric C29S and C32S single-mutant BsTrxA proteins treated with AMS migrated in a single band of ∼12.5kDa (Fig. 2a), indicating that all these single-cysteine mutant BsTrxA molecules had bound one AMS molecule. This showed that all single-cysteine mutant BsTrxA monomers were present in a reduced state. In contrast to the ∼12-kDa species of the single-cysteine mutant BsTrxA proteins, no AMS binding could be observed for the ∼25-kDa species of these mutant proteins. This showed that no free thiol groups are available in the ∼25-kDa species. In fact, the dimer of the C29S mutant protein was resolved upon incubation with AMS. Taken together, these findings strongly support the view that the ∼25-kDa species of the C29S and C32S single-mutant BsTrxA proteins indeed represent dimers of two identical BsTrxA subunits held together by a disulfide bond.

### BsTrxA homodimer structure

**Fig. 3:**
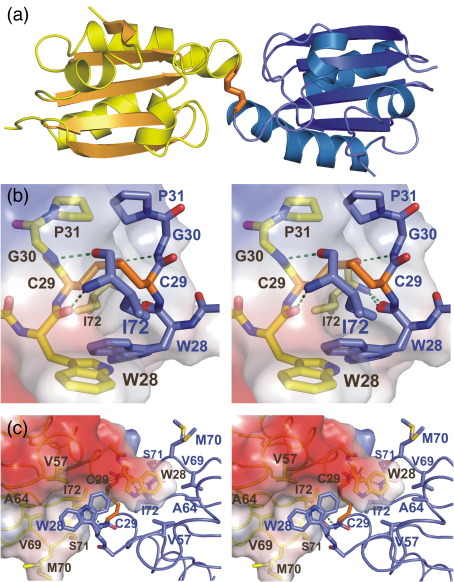
Overall fold and the dimer–interface interactions of the C32S BsTrxA dimer. (a) Overall fold of the C32S BsTrxA dimer. The disulfide-bonded C32S BsTrxA chains are shown in yellow (chain A) and in blue (chain B). The C29–C29 disulfide bond between the chains is shown in orange. (b) Stereo diagram of the active site of the C32S BsTrxA dimer with an electrostatic surface shown for chain A. The C32S active-site residues Trp28, Cys29, Gly30, and Pro31 are shown. Carbon atoms are shown in yellow in chain A and in blue in chain B. Nitrogen and oxygen atoms are in dark blue and red, respectively. The disulfide bond atoms are in orange, and hydrogen bonds are shown in green. (c) The shallow hydrophobic binding sites for the Trp28 residues on the opposite chains of the dimer in the C32S mutant of BsTrxA. The color coding is the same as in (b), and the view is an approximately 90° rotation relative to (b).

In order to verify the suggestion that the C32S BsTrxA protein forms a homodimer linked together via a disulfide bond between the attacking cysteines in the identical subunits, we crystallized this protein and subsequently determined its three-dimensional structure to 1.5Å (Fig. 3a). The crystals belonged to space group *P*1, with unit cell dimensions of *a* = 36.8Å, *b* = 38.4Å, *c* = 41.9Å, α = 83.3°, β = 66.6°, and γ = 78.1° (Table 1). There was one copy of the dimer in the asymmetrical unit. The C32S BsTrxA homodimer has the expected thioredoxin fold, with a central hydrophobic core with a four-stranded β sheet surrounded by five α helices on the external surface (Fig. 3a). The C32S homodimer structure closely resembles the NMR structure,^12^ with an rmsd of 0.9 Å between 86 C^α^ carbon atoms (comparing the first model of the 2IPA heterodimer BsTrxA NMR structure with chain A of the C32S BsTrxA structure). It is also similar to the known human and *E. coli* structures (PDB codes 2TRX, 1XOA, 1XOB, 1ERU, 1ERT, 1CQG, 1CQH, and 1MDJ) with rmsd values of 1–1.5Å for global C^α^ superpositions. This underscores the strong conservation of thioredoxin in nature. The active-site amino acid sequence WCGPC forms a loop linking the end of a β strand (β2) with the beginning of an α helix (α2).

Importantly, C32S TrxA does indeed crystallize as a dimer held together by a disulfide bond between the two active sites attacking Cys29 residues. In addition, dimerization is facilitated by intermolecular hydrogen bonds and hydrophobic interactions. Intermolecular hydrogen bonds occur between the backbone amide nitrogen of Ile72 and the carbonyl of Trp28, and between the backbone carbonyl of Ile72 and the amide nitrogen of Gly30 (Fig. 3b). No side chains are involved in intermolecular bonds in this structure. The large hydrophobic residue Trp28, which precedes the catalytic Cys29, interacts with a shallow hydrophobic pocket on the other chain consisting of the side chains of Ala26, Trp28, Val57, Ala64, Val69, and Ile72 (Fig. 3c). In addition, the backbone of residues Met70 and Ser71 packs against the Trp28 ring (Fig. 3c). The chain B Trp28 has two alternative conformations, whereas the chain A Trp28 has only one orientation. In both chains, the hydrophobic Trp28 interacts with the same surface on the opposite chain. This series of hydrogen bonds and hydrophobic interactions stabilizes the dimer formation.

**Fig. 4:**
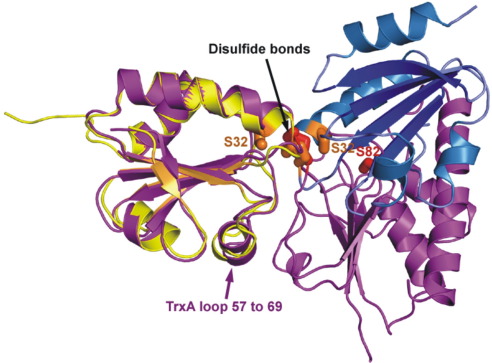
Comparison of the *B. subtilis* TrxA homodimer and BsTrxA–ArsC complex structures. The *B. subtilis* TrxA homodimer structure is shown as a cartoon with chains A and B in yellow and blue, respectively. The disulfide bridge and the Ser32 are shown in orange, as in Fig. 3. The BsTrxA–ArsC complex structure is shown in purple, with the disulfide bridge and residue Ser82 (from the C82S mutation in ArsC) shown in red.

Comparison of the BsTrxA homodimer structure and the TrxA–ArsC heterodimer structure shows that although the fold of BsTrxA is very similar, its binding mode is different (Fig. 4). Both structures show extensive interactions with the catalytic loop between Ala26 and Gly30. They both interact with the loop and helix structure between Val57 and Val69, although the BsTrxA–ArsC structure has a much more extensive interaction. Only ArsC interacts with the loop from residues Val88 to Lys91. Whereas the BsTrxA homodimer uses the backbone amide and carbonyl of Ile57 to form hydrogen bonds to the other chain, the BsTrxA–ArsC structure does not have these interactions. The two complexes therefore show very different binding modes.

### Comparison with thioredoxin complex structures

Mixed disulfide intermediates of thioredoxin have previously been characterized at the structural level by NMR of human thioredoxin with two substrate peptides derived from Ref-1 and nuclear factor κB (NF-κB;^14,15^ PDB codes 1CQG, 1CQH, and 1MDJ), by NMR of the BsTrxA–ArsC complex,^12^ and by X-ray crystallography of barley thioredoxin complexed with the substrate protein, BASI (HvTrxh2–BASI).^16^ In all these complex structures, the resolving cysteine in thioredoxin was mutated to prevent the reaction from proceeding, which would have caused the complex to dissociate.

**Fig. 5:**
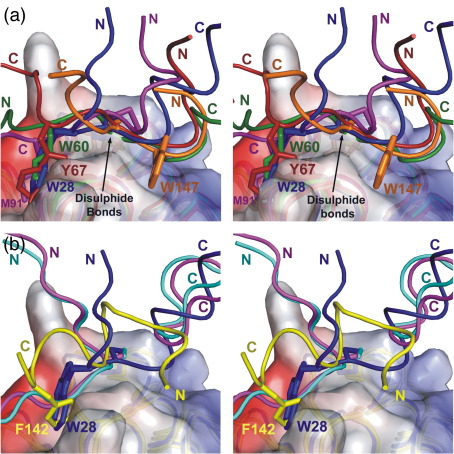
Comparison of protein/peptide binding modes in the BsTrxA C32S dimer and other thioredoxin peptide and protein complexes. (a) BsTrxA was superimposed on the thioredoxin from other complexes using PyMOL.^35^ The superimposed structures, the surface of chain A of BsTrxA, and the peptide or protein chain of the bound molecule are shown. The disulfides and large hydrophobic residues that contribute to binding are also shown. The N- and C-termini of the bound peptides and proteins are indicated with the letters N and C. The proteins shown in (a) are as follows: the BsTrxA dimer with residues 26–38 of chain B shown as cartoons and the side chains of Trp28 and Cys29 shown as sticks (this study; dark blue); the NMR structures of human thioredoxin complexed with substrate peptides from Ref-1 (1CQH; red) or NF-κB (1MDJ; green) with the side chains of residues Cys65 and Tyr67 of the Ref-1 peptide and of residues Trp60 and Cys62 of the NF-κB peptide; the NMR structure of the *B. subtilis* TrxA and ArsC (2IPT; purple) with residues Cys89 and Met91; and the X-ray structure of the complex between barley thioredoxin and the α-amylase serine proteinase inhibitor (2IWT; orange) with residues Trp147 and Cys148. (b) Comparison of the BsTrxA (dark blue) dimer interface with the binding of thioredoxin reductase by *E. coli* thioredoxin (1F6M; yellow) and the X-ray structures of the complexes between spinach chloroplast thioredoxins Trx-f and Trx-m and ferrodoxin–thioredoxin reductase (2PU9 and 2PUK; in cyan and magenta, respectively). Trp28 is shown for the TrxA structure (dark blue), and the *E. coli* thioredoxin reductase structure shows Phe142 (yellow).

The structures of the thioredoxins in these complexes were superimposed on chain A of the BsTrxA homodimer structure, and the protein/peptide binding modes were compared (the thioredoxin complex structures used in this study are listed in Table 2). The directions of the polypeptide chain through the thioredoxin active site are different in the NF-κB peptide complex and in the Ref-1 peptide complex^15^ (Fig. 5a). In the BsTrxA–ArsC and HvTrxh2–BASI complexes, the chain direction is similar to the Ref-1 peptide.^16^ The C32S BsTrxA homodimer structure described here, however, has the same peptide chain orientation as the NF-κB peptide (Fig. 5a). Interactions between thioredoxin and its substrates usually involve three loops from thioredoxin: the catalytic loop (residues 31–35 in *E. coli* Trx) and two additional binding loops (residues 74–76 and 91–93 in the *E. coli* structure^16^). In the BsTrxA homodimer structure described here, only two of these loops are involved in the interactions; the third loop (residues 88–91) does not interact with the other chain of the dimer.

In general, the complexes have large hydrophobic residues on the substrate (either Trp, Tyr, or Met) that interact with a shallow hydrophobic groove: Trp28 in the BsTrxA homodimer, Met91 in the BsTrxA–ArsC heterodimer, Tyr67 in the Ref-1 peptide, Trp60 in the NF-κB peptide, and Trp147 in the HvTrxh2–BASI complex. In four of the structures (the two peptide complexes, the C32S BsTrxA homodimer, and the BsTrxA–ArsC structures), the hydrophobic interactions occur in an equivalent part of the thioredoxin surface (Fig. 5a). In contrast, the hydrophobic interaction between Trp147 in the BASI substrate and HvTrxh2 occurs at a different site (Fig. 5a). In addition, each complex is held together by four to five intermolecular hydrogen bonds, although the details of the individual interactions vary. The diversity of the binding modes observed in these five complexes illustrates how thioredoxin can bind to and react with a variety of different substrates.

Thioredoxin also forms complexes with thioredoxin reductase—the enzyme that converts oxidized thioredoxin back to the active reduced state. Structures of complexes of *E. coli* thioredoxin with thioredoxin reductase,^19^ and also chloroplast thioredoxins (Trx-m and Trx-f) with *Synechocystis* ferredoxin–thioredoxin reductase^20^ are available. These show completely different thioredoxin-binding modes (Fig. 5b). The *E. coli* thioredoxin has relatively little contact between the substrate peptide and the protein, although a large hydrophobic residue of the thioredoxin reductase, Phe142, binds in approximately the same position as the hydrophobic residues in the substrate complexes (Fig. 5b). The *Synechocystis* ferredoxin–thioredoxin reductase complexes have two loops that bind at either end of the substrate-binding site (Fig. 5b). The C32S BsTrxA homodimer complex is therefore more closely related to the thioredoxin/substrate complexes, in particular the NF-κB peptide complex, rather than to the complexes with the reductases.

### The resolving cysteine

**Fig. 6:**
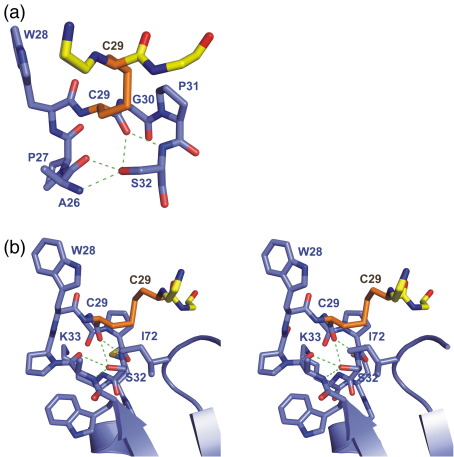
Ser32 interactions in the BsTrxA C32S structure. (a) The loop from residue 26–32 in chain A of the BsTrxA C32 structure is shown with the hydrogen bonds to the Ser32 residue. C29 from chain B is also shown, and the disulfide bond is shown in orange. Oxygen and nitrogen atoms are in red and blue, respectively. The carbon atoms of chains A and B are in blue and yellow, respectively. Hydrogen bonds are shown in green. (b) The environment surrounding S32 is shown. The catalytic loops with residues 25–33 and 72 are shown as sticks, and other parts of the structure are shown as cartoons. The colors are the same as in (a).

The original Cys32 residue in the wild-type BsTrxA would resolve any intermolecular disulfide bonds via its deprotonated sulfhydryl group in the native enzyme. However, in this BsTrxA structure, the C32S mutation is present. The Ser32 mutant is unable to resolve the intermolecular disulfide or form an intramolecular disulfide bond with Cys29. The C32S mutation therefore ensures that the reaction does not proceed along the reaction coordinate. The crystal structure shows that the side chain of the mutated residue C32S points away from the intermolecular disulfide Cys29-Cys29 bond, forming hydrogen bonds to the backbone amide of Ala26 and the carbonyls of Ala26 or Cys29 (Fig. 6a). Similar interactions would not be expected in the wild-type Cys32, allowing the Cys32 side chain to rotate to an alternative rotamer, which would be ideally positioned for attacking the intermolecular disulfide bond intermediate. The Ser32 side chain is packed between the side chains of Trp25, Pro31, and Ile72, so it is buried inside the protein (Fig. 6b). A change of the rotamer of Cys32 from that shown by Ser32 would allow the Cys32 side chain to attack the disulfide bond in the wild-type enzyme. Even with a change in rotamer, Cys32 would not be exposed at the protein surface to the same extent as Cys29. In order for Cys32 to act as the reactive cysteine in the C29S mutant, a change in the conformation of the catalytic loop may be necessary, so that Cys32 could be available on the protein surface to react with substrate proteins.

Taken together, our present structural analysis of the C32S BsTrxA dimer indicates that the high-resolution structure obtained in this study can serve to visualize a mixed disulfide intermediate state in the thiol–disulfide exchange reaction catalyzed by the native enzyme.

## Discussion

In the present study, we characterized active-site mutants of TrxA from *B. subtilis* at the molecular and structural levels. To this end, we highly purified three active-site mutants and the wild-type BsTrxA protein. Mixed disulfide reaction intermediates could be generated, in particular between the C32S BsTrxA and its substrates, but also with the C29S and wild-type BsTrxA. The analyses on our purified BsTrxA proteins revealed that the single-cysteine active-site mutant BsTrxA proteins not only bound substrates but also had a high tendency to bind to each other. Specifically, the pure single-cysteine BsTrxA mutant proteins displayed auto-oxidation of the remaining active-site cysteine residues, which resulted in homodimer formation. We were able to confirm this stable interaction by characterizing the structure of the C32S BsTrxA homodimer at 1.5Å resolution. The structure obtained is unique in that it is the first structure of an active-site cross-linked thioredoxin homodimer that could be regarded as a stable mixed disulfide BsTrxA reaction intermediate. The structure also represents the first example of a thioredoxin protein–protein complex with a chain orientation similar to that of the peptide from NF-κB (Fig. 5a). A common theme observed in all but one of the thioredoxin protein and peptide complexes is an interaction between a shallow hydrophobic groove on the surface of the thioredoxin protein adjacent to the disulphide and a large hydrophobic residue from the binding partner. One complex, the HvTrxh2–BASI complex, lacks this interaction but instead has a site on the other side of the disulphide where a hydrophobic residue is found. These structures therefore illustrate alterative thioredoxin/substrate binding modes.

Our present study shows that both the C32S BsTrxA mutant protein and the C29S BsTrxA mutant protein are able to form mixed disulfide intermediates when provided with potential substrate proteins. To our knowledge, the finding that BsTrxA mutant proteins lacking the attacking cysteine residue can form such intermediates has not been reported before. In fact, most documented interaction profiling studies, based on the mixed disulfide fishing technique, were only performed with a mutated variant lacking the resolving cysteine residue (Cys32 in BsTrxA), but not with mutant forms lacking the attacking cysteine residue (Cys29 in BsTrxA).^29,36,37^ According to the generally accepted model for thioredoxin function, the Cys29 appears as a thiolate, whereas Cys32 is kept protonated.^9,10,38,39^ Our data suggest that the resolving cysteine in the C29S mutant protein is also able to engage in substrate binding. Studies on *E. coli* Trx1 have shown that the unusually low p*K*_a_ of the attacking cysteine is maintained by proton sharing of the thiol proton, which is normally attached to the resolving cysteine, thereby giving the resolving cysteine a high p*K*_a_.^40,41^ Mutation of the attacking cysteine in the C29S protein may therefore result in a lower p*K*_a_ of the resolving cysteine, giving it a higher reactivity towards possible substrates than in the wild-type protein. Our mixed disulfide experiments indicate that Cys29 is more effective in forming stable reaction intermediates with potential substrate proteins than Cys32. In the available BsTrxA C32S mutant structure, the Ser32 side chain is not exposed on the protein surface (Fig. 6b); it is oriented away from the intramolecular disulfide bond and forms a series of hydrogen bonds (Fig. 6a). The wild-type Cys32 would not be expected to form similar hydrogen bonds and may therefore adopt a different position.^12,40^ Rotation of the side chain would allow Cys32 to attack the disulfide bond and complete the reaction. However, a further change in the structure may be required to expose Cys32 on the protein surface, so that it can react with substrate proteins. This change in conformation could perhaps be caused by the C29S mutation, since the serine side chain could form extra interactions that could assist in modifying the structure. This would explain why the C29S mutant BsTrxA does not react as efficiently with substrate proteins relative to the C32S mutant protein. To confirm or falsify this speculation, we would need a high-resolution structure of C29S mutant BsTrxA, which is presently not available.

A second interesting result of the present study was the homodimerization of single-cysteine BsTrxA mutant proteins by thiol oxidation. It should be noted that auto-oxidation of *E. coli* thioredoxin has been mentioned in the literature for a mutant protein lacking the resolving cysteine,^32,33^ but not for a mutant protein lacking the attacking cysteine. Other thioredoxin dimers were shown to be formed by thiol (auto)oxidation of a third cysteine residue outside the active site (e.g., Cys73 of human Trx1).^13^ This Cys73-based dimerization is believed to function as a means to down-regulate thioredoxin activity, since such dimers were found to be inactive.^42^ Notably, the only two-cysteine residues of BsTrxA are the active-site cysteine residues, precluding the possibility of the regulation of thioredoxin activity via oxidation–reduction of a third cysteine residue. Thus, the observed dimerization of the single-cysteine mutant TrxA proteins is unrelated to the regulatory Trx1 dimerization. In addition, dimer formation under reducing acidic conditions via noncovalent binding has been reported for *E. coli* and human thioredoxins.^43–46^ Such thiol-independent dimerization was not observed for the purified BsTrxA. Furthermore, it should be noted that mutants of *E. coli* TrxA with an altered active site (CACA instead of CGPC) were also shown to dimerize via active-site cysteine residues.^47^ In this case, however, the dimerization was due to the fact that the mutation changed the *E. coli* TrxA conformation such that the second cysteine residue of the CACA motif became surface-exposed. Moreover, in this dimer structure, both cysteine residues in the CACA motif were disulfide-bonded. This makes the *E. coli* CACA TrxA dimer quite distinct from the C32S BsTrxA dimer reported here. Altogether, we conclude that the reported dimerization of both single-cysteine active-site mutant BsTrxA proteins by thiol oxidation of the remaining cysteine residues differs substantially from most previously reported mechanisms for thioredoxin dimerization.

It is presently not known whether BsTrxA molecules interact with each other *in vivo*, but this possibility seems likely because both oxidized and reduced forms of BsTrxA can be detected in growing cells.^48^ In any case, the mixed disulfide homodimers of single-cysteine mutant BsTrxA provide an interesting opportunity to study the three-dimensional topology of the normally very short-lived mixed disulfide reaction intermediate state between two proteins taking part in a thiol–disulfide exchange reaction. In the structure of the oxidized C32S BsTrxA mutant dimer, one BsTrxA moiety would represent the enzyme, and the other one would represent the substrate. The structural studies of the human Trx1, in complex with two distinct substrate peptides, showed that the peptides bind in opposite orientations.^14,15^ The structure of C32S BsTrxA described here adopts one of these orientations (that of the NF-κB peptide), whereas the thioredoxin/substrate protein complex HvTrxh2–BASI^16^ and the BsTrxA–ArsC complex^12^ adopt the alternative Ref-1 peptide orientation. The C32S BsTrxA structure therefore represents a different mode by which thioredoxin can bind substrate proteins, demonstrating the existence of the binding mode shown with the NF-κB peptide. The binding of substrate protein is stabilized by hydrogen bonds and hydrophobic interactions between a large hydrophobic group on the binding partner and hydrophobic patches on the surface of thioredoxin (Figs. 3, 5 and 6). The C32S BsTrxA structure, in comparison with other disulfide intermediate structures, therefore illustrates how thioredoxin accommodates a variety of substrates using alternative hydrogen bonding patterns and more than one possible hydrophobic region.

Since the identified C32S BsTrxA homodimer is formed by thiol oxidation between two identical BsTrxA molecules, this implies that these intrinsically reductive molecules undergo auto-oxidation. This is probably due to the fact that our interaction studies were performed *in vitro* under nonreducing conditions. Exposure of the purified proteins to oxidative compounds, such as molecular oxygen, is likely to result in their oxidation. Consistent with this idea, we demonstrated via cross-linking of AMS to free thiols that most of the purified wild-type BsTrxA molecules were in fact oxidized under the conditions used. Thus, the redox state of wild-type BsTrxA *in vitro* may, to some extent, resemble the *in vivo* redox state of this protein.^48^

In conclusion, during the establishment of the necessary tools for mixed disulfide fishing in *B. subtilis*, we discovered that purified single-cysteine BsTrxA mutant proteins are subject to auto-oxidation, forming stable homodimers by oxidation of the remaining cysteine residues. The structure of a C32S homodimer of BsTrxA was solved at high resolution, which provides interesting and novel insights into the BsTrxA active-site geometry during the covalent substrate-bound intermediate state of catalysis. The structure demonstrates a binding mode, which, in comparison with other mixed disulfide intermediate structures, illustrates how thioredoxin can bind to and react with a variety of different substrates.

## Materials and Methods

### Growth conditions and media

Strains were grown with constant agitation (250rpm) at 37 °C in Luria–Bertani medium consisting of 1% tryptone, 0.5% yeast extract, and 1% NaCl (pH7.4). When appropriate, media were supplemented with antibiotics at the following concentrations: chloramphenicol, 5 μg/ml; kanamycin, 50 μg/ml; erythromycin, 2 μg/ml.

### Cloning, expression, and purification of BsTrxA

All procedures for DNA purification, restriction, ligation, agarose gel electrophoresis, and transformation of competent *E. coli* DH5α cells were carried out as previously described.^49^ Chromosomal DNA of *B. subtilis* 168 (*trpC2*)^50^ was isolated according to Bron and Venema, and plasmid DNA was isolated using the High Pure Plasmid Isolation Kit (Roche Applied Science).^51^ The *trxA* gene from *B. subtilis* 168 was PCR-amplified using the primers GGGGGCATATGGCTATCGTAAAAGCAACTGA and GGGGGCTCGAGAAGATGTTTGTTTACAAGCT. The PCR product was ligated into the NdeI and XhoI restriction sites of the pET-26b(+) expression vector (Novagen, Inc.). The resulting pET26-trxA plasmid was subsequently used for site-directed mutagenesis by plasmid PCR with the Extensor PCR mix (ABgene) to obtain mutant *trxA* genes in which codons for Cys were replaced with codons for Ser. Primers C29S-F (GCTCCTTGGTCCGGACCTTGT) and C29S-R (ACAAGGTCCGGACCAAGGAGC) were used to introduce the C29S mutation in BsTrxA; primers C32S-F (TGCGGACCTTCTAAAATGATT) and C32S-R (AATCATTTTAGAAGGTCCGCA) were used to introduce the C32S mutation in BsTrxA; and primers C29S/C32S-F (CCTTGGTCCGGACCTTCTAAAATG) and C29S/C32S-R (CATTTTAGAAGGTCCGGACCAAGG) were used to introduce the C29S–C32S double mutation in BsTrxA. All constructs were checked by sequencing. The generated pET26b plasmids were used to transform *E. coli* BL21(DE3) (Invitrogen) for high-level *trxA* expression and subsequent purification of the wild-type BsTrxA or the different Cys-to-Ser mutant variants of this protein. Notably, all BsTrxA proteins used in the present study contain a C-terminal hexa-histidine His6-tag for purification purposes. This tag is provided by the pET26b(+) vector. For BsTrxA purification, 1-L cultures were grown until an OD_600_ of 0.7 had been reached. Then, IPTG was added to a final concentration of 1 mM to induce BsTrxA production. After 3h of induction, cells were harvested by centrifugation and resuspended in binding buffer [20 mM NaPi, 300 mM NaCl, 10% (vol/vol) glycerol, 5 mM imidazole, and 3mM DTT (pH7.4)]. Next, cells were disrupted by two passages through a French Press (2500 psi). Cellular debris was removed by centrifugation (30 min at 30,000***g*** and 4 °C), and the clarified supernatant fraction was applied to a nickel-charged IMAC column (5 ml of HisTrap HP; GE Healthcare). Unbound sample was washed from the column with binding buffer using an ÄKTA explorer (GE Healthcare). Next, the His-tagged BsTrxA protein was eluted from the column using binding buffer with a gradient of increasing imidazole concentrations (up to 500 mM imidazole). The eluted fractions were checked for the presence of pure BsTrxA protein using SDS-PAGE and subsequent silver staining or Western bloting. Further purification was achieved by concentrating the proteins with Vivaspin columns (Vivascience) and loading them on a Superdex 75 gel filtration column (Amersham) preequilibrated with 20 mM NaPi, 150mM NaCl, 10% glycerol, and 3.5 mM DTT (pH7.4). Fractions containing the purified BsTrxA proteins were pooled and dialyzed three times against 20 mM Tris–HCl (pH7.6) with 150 mM NaCl.

### *B. subtilis* total cytoplasmic proteins

Cytoplasmic proteins were extracted from *B. subtilis* strain WB800 I*trxA* (*trpC2*, *nprE*, *aprE*, *epr*, *vpr*, *bpr*, *mpr*∷*ble*, *nprB*∷*bsr*, *wprA*∷*hyg*; *trxA*∷*pMutin2*; Cm^R^, Em^R^). This strain is based on *B. subtilis* WB800, which lacks eight extracellular proteases,^52^ and it contains a *trxA* gene placed under the transcriptional control of the P*spac* promoter.^23,24^ Thus, protein degradation during the preparation of extracts is minimized and, at the same time, the oxidation of TrxA substrates can be optimized through TrxA depletion. Depletion of TrxA was achieved by growing cells in the presence of 25 μM IPTG.^24^ Cells were harvested from an exponentially growing 1-L culture (OD_600_ = 1.0) and resuspended in 10 ml of protoplast buffer [20 mM potassium phosphate (pH7.5), 15 mM MgCl_2_, and 20% sucrose]. Next, cells were incubated with Complete protease inhibitors at the concentration recommended by the supplier (Roche Applied Science) and 1 mg/ml lysozyme for 30 min at 37 °C, after which the protoplasts were disrupted using a French Press (2500 psi). Cellular debris and the large membrane fraction were removed by centrifugation (30 min, 150,000***g***, 4 °C), and the supernatant fraction containing the cytoplasmic proteins was aliquoted and stored at − 80 °C.

### SDS-PAGE and Western blotting

The presence of TrxA was detected by Western blotting. Proteins were separated by SDS-PAGE (using precast NuPAGE gels from Invitrogen), and proteins were then semi-dry-blotted (75 min at 1 mA/cm^2^) onto a nitrocellulose membrane (Protran®; Schleicher and Schuell). Subsequently, the TrxA proteins were detected with polyclonal antibodies raised against BsTrxA in rabbits (Eurogentec). The detection of bound antibodies was performed with either horseradish-peroxidase-conjugated IgG secondary antibodies and the Super Signal® West Dura Extended Duration Substrate (Pierce) in combination with the ChemiGenius XE Bio-Imaging system (Syngene), or fluorescent IgG secondary antibodies (IRDye 800 CW goat anti-rabbit from LiCor Biosciences) in combination with the Odyssey Infrared Imaging System (LiCor Biosciences). In the latter case, fluorescence at 800 nm was recorded. In addition, protein 50 assay chips, together with the 2100 Bioanalyzer (Agilent Technologies), were used to analyze the proteins. This lab-on-a-chip system allows the separation of proteins by capillary gel electrophoresis. The recorded chromatograms can be converted *in silico* to images that resemble the results of SDS-PAGE. The peak surfaces of the recorded chromatograms were integrated using the 2100 Expert Software (Agilent Technologies) to quantify the relative protein amounts per peak.

### Mixed disulfide fishing

To visualize possible stable interactions between BsTrxA and its substrates, 2 μg of the purified BsTrxA protein variants was mixed with 50 μl of total cytoplasmic protein extract. After 5 min of incubation at room temperature, protein loading buffer was added, and 3 μl of this sample was loaded on a nonreducing gel, and TrxA–substrate-bound proteins were visualized by Western blotting with anti-BsTrxA antibodies.

For purification of the BsTrxA–substrate bound complexes, 40 μg of each purified BsTrxA protein variant was mixed with 250 μl of total cytoplasmic protein extract and left to incubate on ice for 5 min. Next, 30 μl of magnetic beads precharged with nickel (supplied by Zebra Biosciences) was added. The His6-tagged BsTrxA proteins were allowed to bind to the nickel of the magnetic beads for 10min under constant inversion of the tubes. Then, the tubes were placed in a magnetic field until clearing of supernatant had been observed. The clear supernatant was discarded from each tube, and the pellet was washed three times (10 min) with 300 μl of binding buffer [20 mM Tris–HCl (pH7.5) and 300 mM NaCl] under constant tube inversion. Next, the BsTrxA proteins bound to the magnetic beads were washed six times with 400 μl of washing buffer (i.e., binding buffer with 5 mM imidazole). Finally, the tagged BsTrxA proteins with bound potential substrates were separated from the nickel-charged magnetic beads by resuspension in 150 μl of elution buffer (i.e., binding buffer with 300 mM imidazole). The eluted proteins were separated by nonreducing SDS-PAGE, and the presence of TrxA–substrate complexes was visualized by Western blotting with anti-TrxA antibodies.

### Determination of redox states of TrxA monomers and dimers

The purified His6-tagged BsTrxA proteins with wild-type or mutant active sites were analyzed by SDS-PAGE under reducing and nonreducing conditions using precast NuPAGE gels (Invitrogen). Each lane was loaded with ∼2.5 μg of protein. For analysis of BsTrxA proteins under reducing conditions, 10mM DTT was added to the samples prior to SDS-PAGE. In addition, the C32S BsTrxA was analyzed in the presence of increasing concentrations of DTT. Upon electrophoresis, protein bands were stained with Coomassie SimplyBlue™ SafeStain (Invitrogen). The redox state of the purified proteins was monitored by labeling with AMS (Molecular Probes) prior to separation by SDS-PAGE under nonreducing conditions. Notably, AMS will only bind covalently to free thiol groups in reduced BsTrxA molecules, thereby giving the reduced proteins a mass higher than that of the oxidized ones. To quantify the levels of AMS labeling, as well as dimer-to-monomer ratios, the wild-type and mutant BsTrxA proteins were also separated with protein 50 assay chips on a 2100 Bioanalyzer (Agilent Technologies).

### Crystallization

For crystallization, purified protein was buffer-exchanged into a solution containing 25 mM sodium phosphate (pH8.0) and 400 mM NaCl and concentrated to 10 mg/ml. Hanging-drop crystallization experiments were performed with 1 μl each of protein and well solution using 24-well Linbro plates stored at 4 °C. The crystallization solutions and the temperature choice were based on the published conditions used to crystallize TrxA from *E. coli*: 0.1 M Tris (pH8.5), 0.2 M magnesium chloride, 4% acetonitrile, and 30% polyethylene glycol 4000.^47^ Crystals of the C32S single mutant grew in 0.1 M Tris (pH7.8), 0.1 M magnesium chloride, 4% acetonitrile, and 35% polyethylene glycol 4000. The crystals appeared in 1–2weeks with dimensions of approximately 100 μm × 50 μm × 20 μm.

### Data collection and structure determination

Crystals were cryocooled directly into liquid nitrogen, and diffraction data were collected at the European Synchrotron Radiation Facility (ESRF) beamline BM14. Data collection statistics are summarized in Table 1. The data were scaled and processed using the program HKL2000.^53^ Molecular replacement was performed using the program PHASER^54^ in the CCP4 program suite, with a polyserine model of TrxA from *E. coli* (PDB code 2TRX) as the initial model.^18^ After a round of rigid-body and restrained refinement using REFMAC5^55^ from the CCP4 program suite, the model-building program ARP-WARP^56^ was used to build a starting model from the BsTrxA C32S sequence. The model was improved by several rounds of model building and refinement, using the programs Coot^57^ and REFMAC5. Model geometry was analyzed using Coot and the program MolProbity.^58^ The two Pro31 side chains in the two chains of the homodimer are only 2.6 Å apart in the current model. This provides the best fit to the electron density. This clash would not normally occur in the wild-type enzyme. It is likely that the two proline residues adopt alternative nonclashing positions in solution, but these are not apparent in the averaged electron density map and so they are not included in the model. Figures were generated using the program PyMOL.^35^ Structures were superimposed using the align command in PyMOL.^35^

### Accession numbers

Coordinates and structure factors have been deposited in the PDB with accession number 2VOC.

## Figures and Tables

**Table 1:** TrxA C32S data collection and refinement statistics

*Data processing*	
Beamline	ESRF BM14
Wavelength (Å)	0.98
Space group	*P*1
Unit cell	
*a*, *b*, *c* (Å)	36.8, 38.4, 41.9
α, β, γ (°)	83.3, 66.6, 78.1
Resolution (Å)	50.0–1.5
Total observations	86,633
Unique reflections	31,147
Completeness (%)	94.5 (90.1)
Redundancy	2.8 (2.4)
*I*/σ(*I*)	15.6 (1.9)
*R*_sym_ (%)	7.0 (33.9)

*Refinement*	
Resolution (Å)	23.0–1.5
*R*-factor (%)	18.5
*R*_free_ (%)	23.7
rmsd from ideal values	
Bond length (Å)	0.021
Bond angles (°)	2.027
Ramachandran plot	
Most favored regions (%)	98.1
Additional allowed regions (%)	1.9
Generously allowed regions (%)	0.0
Disallowed regions (%)	0.0

Crystals were cryocooled directly into liquid nitrogen, and diffraction data were collected at the ESRF beamline BM14. Data were processed and refined as described in Materials and Methods.

**Table 2:** Thioredoxin complex structures used in this study

PDB code	Thioredoxin (mutation)	Trx catalytic residue: substrate target residue	Other proteins in complex (mutation)	Method	Resolution (Å)	Reference
*Thioredoxin–substrate complexes*						
1MDJ	Human Trx (C35A, C65A, C69A, C73A)	C32 : C62	Peptide consisting of residues 56–68 from the human substrate NF-κB	NMR	N/A	14
1CQG	Human Trx	C32 : C65	Peptide consisting of residues 59–71 from the human substrate Ref-1	NMR	N/A	15
1CQH	(C35A, C65A, C69A, C73A)					
2IWT	Barley HvTrxh2 (C49S)	C46 : C148	α-Amylase serine proteinase inhibitor (BASI) (C144S)	X-ray	2.3	16
2IPA	BsTrxA (C32S)	C29 : C89	ArsC (C10S, C15A, C82S)	NMR	N/A	12
2VOC	BsTrxA (C32S)	C29 : C29	BsTrxA (C32S) homodimer	X-ray	1.5	This study

*Thioredoxin–thioredoxin reductase complexes*						
2PU9	Spinach chloroplast (Trx-f) (C49S)	C46 : C57	*Synechocystis* ferrodoxin–thioredoxin reductase	X-ray	1.65	20
2PUK	Spinach chloroplast (Trx-m) (C40S)	C37 : C57	*Synechocystis* ferrodoxin–thioredoxin reductase	X-ray	3.0	20
1F6M	*E. coli* TrxA (C35S)	C32 : C138	*E. coli* thioredoxin reductase (C135S)	X-ray	3.0	19

*Other thioredoxin complexes*						
1T7P	*E. coli* TrxA	N/A	Bacteriophage T7 DNA polymerase and an DNA strand	X-ray	2.2	21

N/A, not available. Structural data were retrieved from the PDB. The accession codes, relevant mutations, catalytic residues, interacting proteins, and structure analysis details are summarized.
